# Homogeneous microwave near-field power focusing using a cylindrical antenna array

**DOI:** 10.1038/s41598-023-41866-z

**Published:** 2023-09-07

**Authors:** Mohammad-Ali Damavandi, Mohammad Khalaj-Amirhosseini

**Affiliations:** Electromagnetic Waves Propagation Laboratory, School of Electrical Engineering, Iran University of Science and Technology, Tehran, 1684613114 Iran

**Keywords:** Biomedical engineering, Electrical and electronic engineering

## Abstract

This paper presents an investigation of a microwave near-field power focusing (NFPF) analytical approach utilizing a cylindrical array of electric line sources along the z-axis, applicable to any arbitrary homogeneous linear medium. A novel parameter, termed the “focus ability” (FA), is introduced to quantitatively assess a method's capabilities in achieving power focusing. In the following, the phases and amplitudes of the excitation signals of the array elements are obtained with optimization to maximize FA over a certain studied area. Each stage of the theoretical analysis is supported through COMSOL full-wave simulations, ensuring the validity of the results. Theoretical results relevant to focused power are then compared to outcomes obtained from CST full-wave simulations with the same array configuration while employing half-wave dipole antenna elements with excitations obtained via optimization. These comparisons indicate that the excitation signals obtained by theoretical analysis can be used for power focusing when the array of half-wave dipole antenna sources is utilized. Furthermore, the proposed method leads to a significant reduction in the optimization time. Also, in the provided examples, the FA parameter and NFPF are investigated for the different conditions consisting of the type of the homogeneous medium and the number of antennas.

## Introduction

Microwave power focusing is a prominent subject in both theoretical and practical applications. There are several methods for microwave power focusing based on the applications and demands^1,2^. In recent years, due to the increasing number of applications in the near-field, there has been a growing interest among researchers on microwave power focusing in the near-field. Some of these applications include microwave hyperthermia^3–7^, as well as microwave power transfer^8–14^, and radio frequency identification (RFID)^15–18^. Although lenses^10,19,20^, and parabolic antennas can focus the radiation field at their fixed focal point, mainly, focusing employs an array of antennas due to their appropriate flexibility to choose an arbitrary focal point, control the sidelobes level and the shape of the focusing pattern, and implementing the multi-focal points^21^. There are various array arrangements for power focusing, including planar and linear arrays. In near-field power focusing (NFPF), the purpose is to increase the power density in a specific point or area near the antenna, while it is minimum in the other size-limited studied area. Since the focusing of the electromagnetic field can lead to an increase in the density of the electromagnetic power, one can call NFPF also near-field focusing (NFF). Indeed, the fundamental concept behind the focusing methods is the proper selection of the excitation signal of each antenna in an array^1^. The main methods used to implement this idea consist of two techniques: the first one involves phase compensation or conjugate phase (CP)^1,22,23^ and the other one utilizes optimization^24–30^. Additionally, the time reversal method^3,5,6^ is a wideband approach that can be considered as the counterpart of the phase conjugate method. Although CP methods are the simplest focusing techniques for a single focal point, a suboptimal solution for multiple foci is proposed in^22^. In the CP method, due to the predominantly equal excitation amplitudes of the array elements, there is limited control over the sidelobes. In^23^, the combination of a Dolph–Chebyshev amplitude taper with the conjugate phase (CP) method is employed to control the sidelobe level (SLL) of the electric field in the focal plane. In optimization methods, the excitation signals of the array elements are determined in a manner that optimizes a specific parameter according to the proposed method in the article. In^24^, the excitation signals of array elements are computed based on the geometry of the array, by maximizing the power transmission efficiency (PTE) parameter and its extended versions. This method allows for various power distributions and multi-focal points. However, it requires knowledge of the scattering parameters of the system.

In^28^, a comprehensive study is conducted to synthesize the near-field and optimize the radio frequency focusing performance in the near-field. The work introduces an energy-based matrix representation that allows derivation of a concise matrix form describing the contributions of radiation, loss, and reactive power. This approach results in eigenmodes and eigenfields of a linear multi-port network and antenna array, which fully describe all the energy-related processes occurring within the considered structure. In^29^, field synthesis techniques are presented, according to the previous work in^28^, which utilize radiation matrix eigenfields of multi-port antennas for synthesizing near-field distributions. These techniques synthesize fields on both open and closed surfaces and then are applied to synthesize target fields, which can be expressed in terms of both E and H fields or only the E field. In^30^, two synthesis methods for the excitations of near-field (NF) arrays have been introduced, building upon the research on the EM inner product and eigenmode expansions of the radiated fields in^24^ and^28^. These methods involve defining an inner product on the electromagnetic fields and are referred to as the “maximum norm” and “minimum error field norm” methods. The “maximum norm” method, utilizing the inner product, identifies the array excitations that maximize the power flow through a target surface. The “minimum error field norm” method aims to synthesize a given target field in a specific region by driving the excitation vector that radiates the closest EM field to the target field, while minimizing the norm of the error field.

Planar and linear microstrip arrays are among the technologies utilized for implementing NFPF, requiring a feeding network to set the proper phases^1^. Moreover, tansmitarrays^18^ and reflectarrays^31,32^ are used for this purpose, but employing geometrical changes in each unit cell to apply phase compensation^1^. Metasurfaces and dispersion engineering techniques have recently offered notable utility in near-field focusing techniques. They enable effective manipulation of propagating waves, and allowing for the control and tuning of near-field wave characteristics, such as achieving multi-focus characteristics in wireless power transfer^33–35^.

Another approach of NFPF implementation include utilizing of an array of leaky waves and slotted waveguide antennas^36^. Linear arrays around the circumferential of the studied area, often forming a circular arrangement, are typically used in hyperthermia or some of the RFID applications to focus the power at a desired point in the study area. For these structures, a separate unit acts as a feeding network, providing the excitation signals with the desired phases to feed the antennas.

In this paper, a comprehensive analytical investigation of the near-field electromagnetic power for a circumferential array of electric line sources has been conducted, and the results are validated using COMSOL Multi-Physics^37^ full-wave simulations. In the following section, a new parameter called the focus ability (FA) is introduced to evaluate the capability of a method in focusing the desired parameter within an interested part of the studied area. Next, the optimal phase and amplitude values of the excitation signals of the electric line sources, serving as the array elements, are determined through the utilization of optimization techniques. The objective is to maximize the FA value to achieving efficient microwave power focusing at the desired point. In each theoretical result relevant to focused power, the obtained excitations from the theory have been applied in CST Studio^38^ full-wave simulations for the same array configuration with half-wave dipole antenna elements with the sinusoidal input currents at a specific frequency. Comparing the observed results indicates that the power focusing behavior is very similar for both cases, suggesting that the obtained excitations using theoretical analysis can serve as a good estimation for the half-wave dipole excitations, enabling efficient power focusing at the desired point. Consequently, the analytical results can be used instead of a full-wave to solve the optimization problem which leads to great increases in the speed of optimization.

## Method and problem statement

In some applications such as non-invasive hyperthermia using microwaves, and RFID, an array of antennas is usually used around the target and the studied area. The arrangement of the antennas in a circumferential array around the studied area enables us to focus the electric field and power at the desired location by selecting the appropriate amplitudes and phases of the antenna excitations.

In this paper, the studied area is treated as a homogeneous medium with no target matter, and the properties of the medium in the studied area are assumed to be the same as the background medium.

In this scenario, a cylindrical array of linear electric sources, treated as antennas, is positioned around the studied area. The objective is to achieve power focusing at a specific point within the area by selecting the appropriate amplitudes and phases of the excitation signals of the array's elements. The simplest possible case is to consider the medium as a free space or a simple space with arbitrary permittivity and conductivity. Here, linear electric sources are used in the theoretical analysis and COMSOL full-wave simulations, and half-wave dipole antennas are used in the CST full-wave simulations.

**Figure 1:**
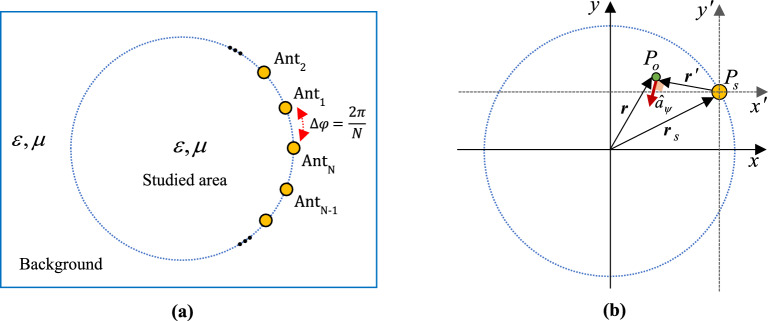
The two-dimensional geometry of the cylindrical arrangement of the antenna array in the $z = 0$ plane. (**a**) The array consists of $N$ similar antennas that are uniformly placed within a homogeneous medium. The background and the studied area have the same medium type. (**b**) Demonstration of $\hat{a}_{\psi }$ for the observation point of $P_{o}$ and one of the sources placed at the point of $P_{s}$. As can be seen, $\hat{a}_{\psi }$ is perpendicular to ${\varvec{r}}^{\prime }$, which is the observation point vector in the $x^{\prime } y^{\prime } -$ coordinate.

Assuming $N$ similar antennas (${\rm Ant}_{i} , \, i = 1, \ldots, N$) are uniformly placed around the studied area, each at the same distance from each other. If the hypothetical region is considered as an infinite cylinder, in the two-dimensional view, each of the antennas will be placed on the circle around the studied area with the same angular distance ($\Delta \varphi = {{2\pi } \mathord{\left/ {\vphantom {{2\pi } N}} \right. \kern-0pt} N}$), as shown in Fig. 1a. Also, $\hat{a}_{{\psi_{i} }}$ is equivalent to $\hat{a}_{{\varphi_{i} }}$ when the position of the *i*-th source is considered as the origin of coordinates as shown in Fig. 1b. The electric and magnetic fields at a certain frequency for the *i*-th source can be expressed as follows^39,40^:

1 $${\mathbf{E}}_{i} (\rho ,\varphi ,z) = E_{{z_{i} }} \hat{a}_{z} = - I_{{e_{i} }} \frac{{k^{2} }}{4\omega \varepsilon }H_{0}^{(2)} (kR_{i} ) \, \hat{a}_{z} ,\;{\text{and }}i = 1, \ldots , \, N$$

2 $${\mathbf{H}}_{i} (\rho ,\varphi ,z) = H_{{\psi_{i} }} \hat{a}_{\psi_{i} } = - jI_{{e_{i} }} \frac{k}{4}H_{1}^{(2)} (kR_{i} ) \, \hat{a}_{\psi_{i} } ,\,{\text{and}}\,i = 1, \ldots , \, N$$

where, $I_{e}$ represents the excitation current amplitude, while $k = \omega \sqrt {\mu \varepsilon } \,$, $\varepsilon { = }\varepsilon^{\prime } - j\varepsilon^{\prime \prime }$, and $\mu { = }\mu^{\prime } - j\mu^{\prime \prime }$ respectively denote the wavenumber, permittivity, and permeability of the arbitrary homogeneous linear medium. $H_{0}^{(2)}$ and $H_{1}^{(2)}$ are Hankel functions of the second kind with orders zero and one, respectively. Additionally, $R_{i}$ is the distance of the observation point from the *i*-th source, calculated as follows:

3 $$R_{i} = \left| {{\varvec{r}} - {\varvec{r}}_{{s_{i} }} } \right| = \sqrt {\left( {x - x_{{s_{i} }} } \right)^{2} + \left( {y - y_{{s_{i} }} } \right)^{2} }$$

where ${\varvec{r}}$ and ${\varvec{r}}_{{s_{i} }}$ respectively represent the observation point and the *i*-th source location vectors from the origin, and $\left( {x,y} \right)$ and $\left( {x_{{s_{i} }} ,y_{{s_{i} }} } \right)$ denote the coordinates of the observation point and the *i*-th source in the $xy -$ plane. The electric and magnetic fields for the general case, taking into account all sources, can also be expressed as follows using relations (1), (2), and (3):

4 $${\mathbf{E}}_{Total} = {\rm E}_{z - Total} \, \hat{a}_{z} = \sum\limits_{i = 1}^{N} {E_{{z_{i} }} } \hat{a}_{z} = - \frac{{k^{2} }}{4\omega \varepsilon }\sum\limits_{i = 1}^{N} {\left( {I_{{e_{i} }} H_{0}^{(2)} (kR_{i} )} \right)\hat{a}_{z} }$$

5 $${\mathbf{H}}_{Total} = \sum\limits_{i = 1}^{N} {H_{{\psi_{i} }} \hat{a}_{{\psi_{i} }} } = - j\frac{k}{4}\sum\limits_{i = 1}^{N} {\left( {I_{{e_{i} }} H_{1}^{(2)} (kR_{i} )\hat{a}_{{\psi_{i} }} } \right)} {\mathbf{ = }}{\rm H}_{x - Total} \hat{a}_{x} + {\rm H}_{y - Total} \hat{a}_{y}$$

By utilizing Eqs. (2), and (5), the total magnetic field in the $\hat{a}_{x}$ and $\hat{a}_{y}$ direction can be formulated as follows:

6 $${\rm H}_{x - Total} = \sum\limits_{i = 1}^{N} {H_{{\psi_{i} }} \alpha_{i} } = - j\frac{k}{4}\sum\limits_{i = 1}^{N} {\left( {I_{{e_{i} }} H_{1}^{(2)} (kR_{i} )\alpha_{i} } \right)} \,$$

7 $${\rm H}_{y - Total} = \sum\limits_{i = 1}^{N} {H_{{\psi_{i} }} \beta_{i} } = - j\frac{k}{4}\sum\limits_{i = 1}^{N} {\left( {I_{{e_{i} }} H_{1}^{(2)} (kR_{i} )\beta_{i} } \right)} \,$$

where the values of $\alpha_{i}$ and $\beta_{i}$ are calculated using the following relations, which are obtained in Appendix (1) in the supplementary material, such that $\hat{a}_{{\psi_{i} }} = \alpha_{i} \hat{a}_{x} + \beta_{i} \hat{a}_{y}$:

8 $$\alpha_{i} = - \frac{{\left( {y - y_{{s_{i} }} } \right)}}{{R_{i} }},\;\beta_{i} = \frac{{\left( {x - x_{{s_{i} }} } \right)}}{{R_{i} }}$$

**Figure 2:**
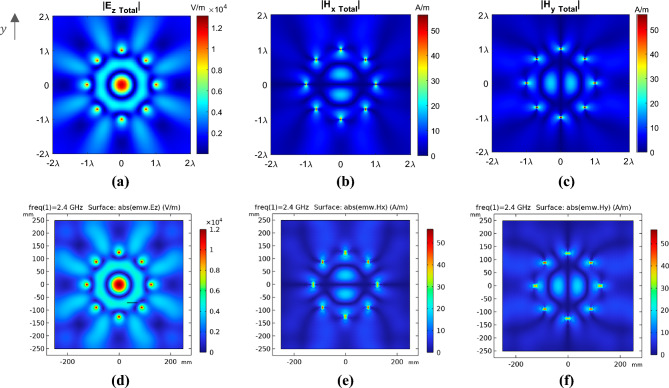
The two-dimensional view of the total electric and magnetic fields for the cylindrical array of antennas in the free space for $N = 8$, $f = 2.4\,{\rm GHz} \,$, and $I_{{e_{i} }} = 1$ in the $z = 0$ plane. The absolute values of the amplitudes for (**a**,** d**) ${\rm E}_{{z - {\rm Total}}}$, (**b**,** e**) ${\rm H}_{{x{- \rm Total}}}$, and (**c**,** f**) ${\rm H}_{{y-{\rm Total}}}$ are obtained using, theoretical analysis in the first row, and COMSOL full-wave simulations in the second row.

Figure 2 demonstrates the total electric and magnetic fields obtained from the analytical analysis and the COMSOL full-wave simulations using the same excitation for the electric line sources.

### Near-field electromagnetic power

Generally, the complex power density flux of electromagnetic waves within a considered region, in this case, the cylinder surrounded by the antenna array and its background, can be calculated using the complex Poynting vector as follows^39,40^:

9 $${\mathbf{S}}\,{ = }\,\frac{1}{2}{\mathbf{E}}{ \times }{\mathbf{H}}^{*} \,{ = }\,\frac{1}{2}{\mathbf{E}}_{{{Total}}} { \times }{\mathbf{H}}_{{{Total}}}^{*}$$

By substituting Eqs. (4) and (5) into the above relation, the Poynting vector can be expressed as follows:

10 $${\mathbf{S}} = \frac{1}{2}\left( {\sum\limits_{j = 1}^{N} {{E}_{{z_{j} }} } \hat{a}_{z} } \right) \times \left( {\sum\limits_{i = 1}^{N} {{H}_{{\psi_{i} }} \hat{a}_{\psi_{i} } } } \right)^{ * } = {S}_{x} \hat{a}_{x} + {S}_{y} \hat{a}_{y}$$

By simplifying Eq. (10), the components ${S}_{x}$ and ${S}_{y}$, which respectively represent the Poynting vector components in the direction of $\hat{a}_{x}$ and $\hat{a}_{y}$ for an arbitrary homogeneous medium, can be obtained as follows. The detailed derivations can be found in Appendix (2) of the supplementary material.

11 $${S}_{x} = j\frac{{k^{2} }}{32\omega \varepsilon }k^{*} \sum\limits_{j = 1}^{N} {\sum\limits_{i = 1}^{N} {I_{{e_{j} }} I_{{e_{i} }}^{*} H_{0}^{(2)} (kR_{j} )\left( {H_{1}^{(2)} (kR_{i} )} \right)^{*} \beta_{i} } }$$

12 $${S}_{y} = - j\frac{{k^{2} }}{32\omega \varepsilon }k^{*} \sum\limits_{j = 1}^{N} {\sum\limits_{i = 1}^{N} {I_{{e_{j} }} I_{{e_{i} }}^{*} H_{0}^{(2)} (kR_{j} )\left( {H_{1}^{(2)} (kR_{i} )} \right)^{*} \alpha_{i} } }$$

**Figure 3:**
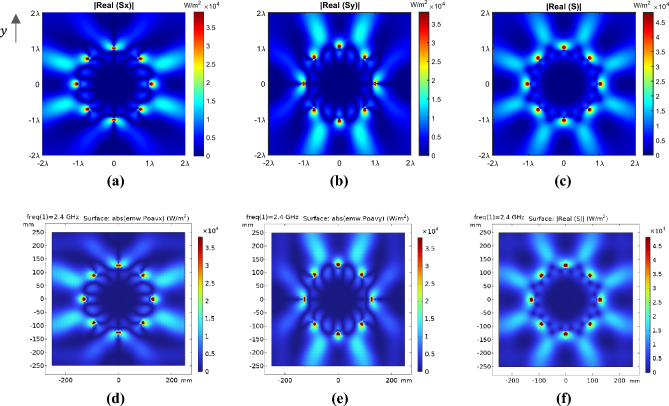
The two-dimensional view of the Poynting vector for the cylindrical array of antennas in the free space for $N = 8$, $f = 2.4 \, {\rm GHz} \,$, and $I_{{e_{i} }} = 1$ in the $z = 0$ plane. The absolute value of the real part of ${\rm S}_{x}$ and ${\rm S}_{y}$, as well as ${\mathbf{S}}$, for (**a**–**c**) theoretical analysis and (**d**–**f**) COMSOL full-wave simulations, respectively.

As depicted in Fig. 3, the real parts of the complex Poynting vector and its components in relations (11) and (12) are verified with full-wave simulation. As we know the real part of the complex Poynting vector is the same as the time average of the instantaneous Poynting vector^39,40^.

The volume density of power leaving a point is expressed by the divergence of the Poynting vector at that point, as follows:

13 $$p_{f} = \nabla . \, S = \frac{{\partial S_{x} }}{\partial x} + \frac{{\partial S_{y} }}{\partial y}$$

By substituting relations (11) and (12) in relation (13) and simplifying it as detailed in Appendix (3) in the supplementary material, the volume density of power leaving a point, for an arbitrary homogeneous medium is obtained as follows:

14 $$p_{f} = j\frac{{k^{2} }}{32\omega \varepsilon }k^{*} \sum\limits_{j = 1}^{N} {\sum\limits_{i = 1}^{N} {\left( {I_{{e_{j} }} I_{{e_{i} }}^{*} \left( {H_{0}^{(2)} (kR_{j} )\left( {kH_{0}^{(2)} (kR_{i} )} \right)^{*} - kH_{1}^{(2)} (kR_{j} )\left( {H_{1}^{(2)} (kR_{i} )} \right)^{*} \left( {\beta_{j} \beta_{i} + \alpha_{j} \alpha_{i} } \right)} \right)} \right)} }$$

**Figure 4:**
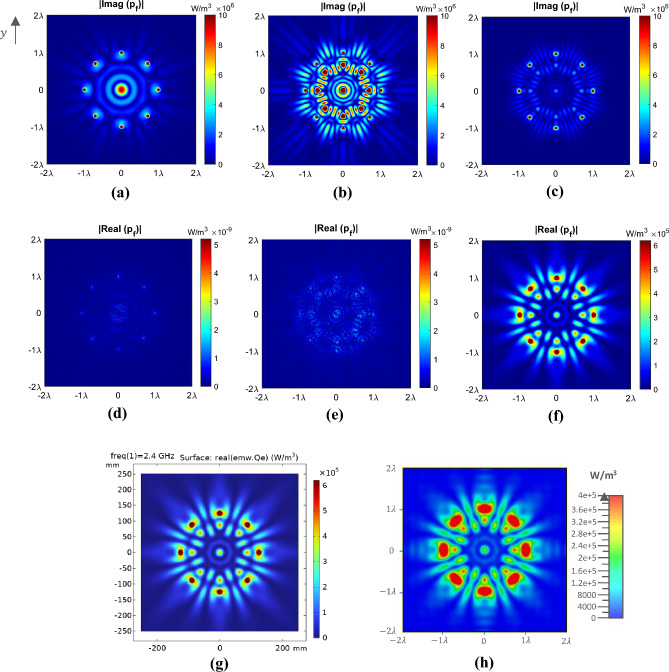
The two-dimensional view of $p_{f}$ for the cylindrical array of antennas with $N = 8$, $f = 2.4\,{\rm GHz} \,$, and $I_{{e_{i} }} = 1$ in the $z = 0$ plane. The absolute value of the imaginary part of $p_{f}$, (**a**) free space, (**b**) $\varepsilon_{r} = 5.28$, and (**c**) $\varepsilon_{r} = 5.28$, $\sigma = 0.104$. The absolute value of the real part of $p_{f}$(**d**) free space, (**e**) $\varepsilon_{r} = 5.28$, and (**f**) $\varepsilon_{r} = 5.28$, $\sigma = 0.104$. (**g**) COMSOL full-wave simulation and (**h**) CST full-wave simulation for a medium with $\varepsilon_{r} = 5.28$, $\sigma = 0.104$.

The results of the theoretical analysis are depicted in Fig. 4a–f. Notably, in cases (d) and (e), the real part of the power $p_{f}$ is zero for free space and a dielectric medium without conductivity, while cases (a) and (b) represent their imaginary part. But, in case (f) involving a dielectric medium with conductivity, a tangible power value is observed in the real part of the power $p_{f}$, which is the dissipated power. To verify the theoretical results, the COMSOL full-wave simulation is presented in Fig. 4g. As depicted, both simulations are completely aligned with each other. Subsequently, the excitations derived from the theoretical analysis were employed as the excitations in the half-dipole antenna array. The outcome of the CST full-wave simulation of this array for a conductive dielectric medium is presented in Fig. 4h. As illustrated in this case, the observed power pattern closely resembles the obtained behavior from the theoretical analysis.

On the other hand, the complex differential form of the power conservation equation can be written as follows^40^:

15 $$p_{s} = p_{f} + \overline{p}_{d} + 2j\omega \left( {\overline{w}_{m} - \overline{w}_{e} } \right)$$

where $p_{s}$ is the complex power density supplied by the sources, which has a nonzero value only in the locations of the sources and is zero in the other places. Also, $\overline{p}_{d}$, $\overline{w}_{m}$, and $\overline{w}_{e}$ are the time-average dissipated power density and the time-average electric and magnetic energy densities which can be obtained as follows for any arbitrary homogenous linear medium:

16 $$\overline{p}_{d} = \frac{1}{2}\left\{ {\left( {\sigma + \omega \varepsilon^{\prime\prime}} \right)\left| {{\mathbf{E}}_{Total} } \right|^{2} + \omega \mu^{\prime\prime}\left| {{\mathbf{H}}_{Total} } \right|^{2} } \right\} = \frac{1}{2}\left\{ {\left( {\omega \varepsilon^{\prime} \times \tan \delta } \right)\left| {{\mathbf{E}}_{Total} } \right|^{2} + \omega \mu^{\prime\prime}\left| {{\mathbf{H}}_{Total} } \right|^{2} } \right\}$$

17 $$\overline{w}_{e} = \frac{1}{4}\varepsilon^{\prime}\left| {{\mathbf{E}}_{Total} } \right|^{2}$$

18 $$\overline{w}_{m} = \frac{1}{4}\mu^{\prime}\left| {{\mathbf{H}}_{Total} } \right|^{2}$$

where $\varepsilon^{\prime } = \varepsilon_{0} \varepsilon_{r}$ and $\mu^{\prime } = \mu_{0} \mu_{r}$, which are the same as $\varepsilon$ and $\mu$ in the simple medium. Also, $\tan \delta = {{\left( {\sigma + \omega \varepsilon^{\prime \prime } } \right)} \mathord{\left/ {\vphantom {{\left( {\sigma + \omega \varepsilon^{\prime \prime } } \right)} {\omega \varepsilon^{\prime}}}} \right. \kern-0pt} {\omega \varepsilon^{\prime}}}$ represents the loss tangent for the lossy dielectric.

## Power focusing at an arbitrary point

In the proposed method, it is possible to focus the power at any arbitrary point in the studied area. For this purpose, first, we define the FA parameter and then we obtain the phases of the array elements by optimization such that the FA parameter is maximized at the desired focus point. Also, by using the optimization and the FA parameter, the excitation amplitude of the array elements can be obtained so that the side lobes have the desired behavior. Side lobes here mean any unwanted local maximums of power levels appearing outside the focal point within the studied area. We use $p_{f}$ to define FA and consequently for optimization because it can consider both **E** and **H** fields simultaneously for any homogeneous medium, and covers the power created by both of them as given in Eqs. (15)–(18). Also, in the optimization process, power can be directly optimized regarding FA in specific desired points within the studied area. In this study, due to the utilization of a smaller number of variables in the optimization process, and the significant role that phases play in determining the focus location, especially in single focusing points, the preference is to optimize the phases first and then proceed to optimize the amplitudes. Additionally, simultaneous optimization of both amplitudes and phases appears to be effective for scenarios involving multi-focusing points.

### Definition of focus ability (FA)

A common approach for quantifying the rate of heat generated by absorbed energy in applications like hyperthermia is to utilize the specific absorption rate (SAR) parameter^3–6^. Consequently, focusing the SAR parameter can somehow indicate the focusing of the energy or electromagnetic power. However, the calculation of SAR requires a lossy medium and must be performed in a three-dimensional volume. In this section, a parameter has been introduced to assess the extent of electromagnetic power focusing. This parameter determines the ability level of a method to concentrate the desired power at a desired focusing point in the studied area and is referred to as the “Focus Ability” (FA). Since in this paper, we aim to focus the power $p_{f}$, the FA parameter for two-dimensional power focusing on a certain studied area surface can be defined as follows:

19 $$FA = \frac{{p_{o} }}{{p_{av} }}$$

where $p_{o}$ is $p_{f}$ in the focusing point, and $p_{av}$ is the average of $p_{f}$ over the whole of the studied area with ${\varvec{r}} \le 0.95{\varvec{r}}_{s}$ and therefore the sources are not considered. In other words, FA is the ratio of the desired power value at the focusing point to its average over a certain studied area. Maximizing FA makes it possible to simultaneously maximize the value of power $p_{f}$ at the desired focusing point while minimizing the average power value for the studied area which is the same as minimizing sidelobes. Depending on the matter of the medium the effect of **E** field and **H** field in the power will be different, for example for the considered medium in Fig. 4 the power is mainly affected by **E** field and they have the same behavior, resulting in a focused **E** field. In contrast to SAR^3–6^, FA can be defined in a two-dimensional space independent of the type of medium. Also, for FA, one can define FA_− 3 dB_ or (half power focus ability) HPFA area, which means the area around the desired point where the amount of focused power is reduced by 3 dB or reaches half.

### Phase optimization

In order to focus the power at the desired point, the phase values of the array elements are obtained using an optimization method so that the FA parameter is maximized. Two examples are considered to perform the optimization and the power focusing at $\left( {\rho ,\varphi } \right) = \left( {{\lambda \mathord{\left/ {\vphantom {\lambda 2}} \right. \kern-0pt} 2},{\pi \mathord{\left/ {\vphantom {\pi 3}} \right. \kern-0pt} 3}} \right)$. The first example involves the use of an array with 8 antennas, and the second one utilizes an array with 16 antennas. For both examples, the free space and a lossy dielectric medium with electromagnetic specification near fatty tissue^41^ with $\varepsilon_{r} = 5.28$ and $\tan \delta = 0.145$ at 2.4 GHz, are studied. The gradient-based optimization function in MATLAB^42^, “fmincon” is utilized for the optimization process.

**Figure 5:**
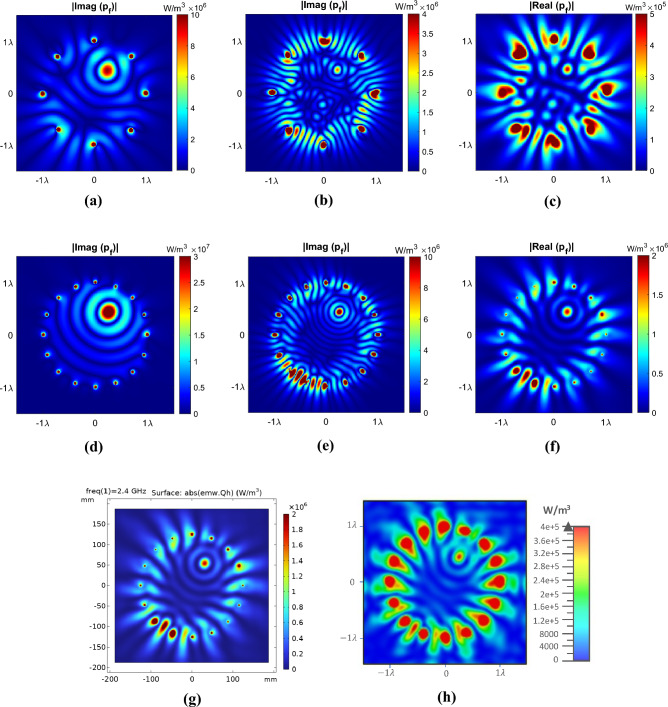
The two-dimensional view of focused power $p_{f}$ at $\left( {\rho ,\varphi } \right) = \left( {{\lambda \mathord{\left/ {\vphantom {\lambda 2}} \right. \kern-0pt} 2},{\pi \mathord{\left/ {\vphantom {\pi 3}} \right. \kern-0pt} 3}} \right)$ for the cylindrical array is presented using (**a**–**f**) MATLAB theoretical, (**g**) COMSOL full-wave, and (**h**) CST full-wave (using an array of half-wave dipole antennas) simulations. This figure shows the absolute value of the imaginary and real parts of $p_{f}$ with $f = 2.4\,{\rm GHz}$, and $|I_{{e_{i} }}| = 1$ in the $z = 0$ plane. Specifically, (**a**–**c**) represent $N = 8$, while (**d**–**g**) represent $N = 16$. Among these, (**a**) and (**d**) depict free space conditions, while (**b**) and (**e**–**h**) illustrate the situation involving a lossy dielectric with $\varepsilon_{r} = 5.28$ and $\tan \delta = 0.145$.

The operation frequency is set at 2.4 GHz, and the current amplitudes are assumed to be equal to one ($|I_{{e_{i} }}| = 1$). The optimized phase values for the examples are provided in Tables 1 and 2. Additionally, the simulation results using the obtained phase values are presented in Fig. 5.

**Table 1:** The obtained optimum phase values for the first example ($N = 8$).

Antenna number	1	2	3	4	5	6	7	8
Antenna Phase [rad] (free space)	0.9999	− 1.1354	− 0.6666	1.8623	− 2.4858	− 1.4945	− 1.7207	− 3.0410
Antenna phase [rad] (the lossy dielectric)	1.7924	3.1929	− 8.1667	3.6789	− 4.0863	4.3353	− 2.3287	0.6953

**Table 2:** The obtained optimum phase values for the second example ($N = 16$).

Antenna number	1	2	3	4	5	6	7	8
Antenna phase [rad] (free space)	− 2.6377	2.3244	− 4.9225	1.2128	1.9790	− 3.1029	− 1.9132	5.3916
Antenna phase [rad] (the lossy dielectric)	0.3377	− 2.5105	1.7361	1.3893	2.9822	− 0.6907	2.1140	− 1.4912

Antenna number	9	10	11	12	13	14	15	16
Antenna phase [rad] (free space)	0.1095	0.7835	1.0716	1.2259	0.9362	0.2896	− 0.5049	− 1.5012
Antenna phase [rad] (the lossy dielectric)	0.6894	− 4.1156	2.9692	2.9046	− 3.8050	1.3084	− 0.7533	3.0206

It is worth noting that the obtained phase values can be expressed as the phase difference with respect to a reference phase, which can be considered as the phase of any of the antennas. These phase differences indeed play a crucial role in determining that the emitted waves have the same phase at the focusing point, resulting in constructive interference. Therefore, the phase vector can be changed by adding or subtracting any offset, and individual phases can be altered by $\pm 2\pi$. Exploiting these characteristics, it becomes apparent that the ratio of obtained phase values for two different mediums, dielectric loss and free space, is approximately equivalent to the ratio of ${{real(k)} \mathord{\left/ {\vphantom {{real(k)} {k_{0} }}} \right. \kern-0pt} {k_{0} }}$ for both arrays, whether they comprise 8 or 16 antennas.

### Amplitude optimization utilizing the optimum phases

**Figure 6:**
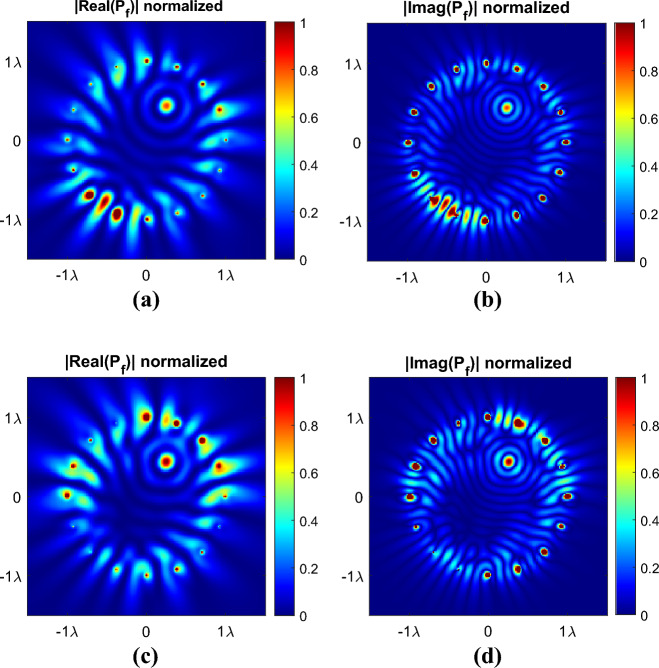
The two-dimensional view of normalized focused power $p_{f}$ at $\left( {\rho ,\varphi } \right) = \left( {{\lambda \mathord{\left/ {\vphantom {\lambda 2}} \right. \kern-0pt} 2},{\pi \mathord{\left/ {\vphantom {\pi 3}} \right. \kern-0pt} 3}} \right)$ for the cylindrical array using MATLAB theorical simulation with obtained phases through optimization. The absolute value of the imaginary and real parts of $p_{f}$ with $N = 16$, and $f = 2.4\,{\rm GHz}$, and $.$ in the $z = 0$ plane for the lossy dielectric with $\varepsilon_{r} = 5.28$ and $\tan \delta = 0.145$. (**a**) and (**b**) depict the scenario with equal amplitudes set to one. (**c**) and (**d**) represent the obtained amplitudes through optimization. FA = 14.1 dB for phase-only optimization, and FA = 14.5 dB for combined phase and amplitude optimization.

As mentioned before amplitudes significantly influence the formation of side lobes, while phases hold a critical role in pinpointing the location of the focusing point. As a result, the process of selecting optimal phases and amplitudes is divided into two distinct, yet interconnected steps. By addressing these elements separately, one can fine-tune the behavior of the array to achieve the desired performance characteristics. The initial step involved obtaining phase values by maximizing FA to ascertain the focus point's location, as explained in the preceding section. The subsequent step pertains to acquiring amplitude values that align with desired conditions for both the focal point and side lobes. Here, the amplitudes are obtained in a manner that maximizes the ratio of the power value at the focusing point to the maximum power value in the area outside of FA_− 3 dB_ within the studied area with ${\varvec{r}} \le 0.95{\varvec{r}}_{s}$. The amplitude values, obtained through genetic algorithm optimization (detailed in Appendix 4 in the supplementary material), for an array with 16 antennas in a lossy dielectric medium with $\varepsilon_{r} = 5.28$ and $\tan \delta = 0.145$, at a frequency of 2.4 GHz, with a focusing point of $\left( {\rho ,\varphi } \right) = \left( {{\lambda \mathord{\left/ {\vphantom {\lambda 2}} \right. \kern-0pt} 2},{\pi \mathord{\left/ {\vphantom {\pi 3}} \right. \kern-0pt} 3}} \right)$, are presented in Table 3. Additionally, the simulation results utilizing the acquired amplitudes and phase values are shown in Fig. 6. The values in this figure are normalized to facilitate comparison between the optimized amplitudes and the case where all amplitudes are uniform and set to one.

**Table 3:** The obtained optimum amplitude values for the second example ($N = 16$).

Antenna number	1	2	3	4	5	6	7	8
Antenna amplitude in the lossy dielectric	0.8219	0.6978	0.8877	1.0000	0.8267	0.4515	0.7759	0.9617

Antenna number	9	10	11	12	13	14	15	16
Antenna amplitude in lossy dielectric	0.8409	0.3970	0.2079	0.2480	0.6468	0.7183	0.6991	0.5586

## Discussion

By employing optimization, it becomes feasible to not only determine the desired location for power focusing but also to exert control over the values of the focused power and sidelobes. Furthermore, the potential for having multiple focusing points is also achievable. It’s important to highlight that the proposed method is particularly effective when the focusing point lies within an area surrounded by array elements. However, it is not suitable for focusing outside of this environment. In such cases, a planar or linear array would be preferable. In the following of this section, we present the reduction in optimization time facilitated by the proposed method and explore variations in the FA parameter in response to changes in the number of antennas and the type of medium.

### Optimization time

**Figure 7:**
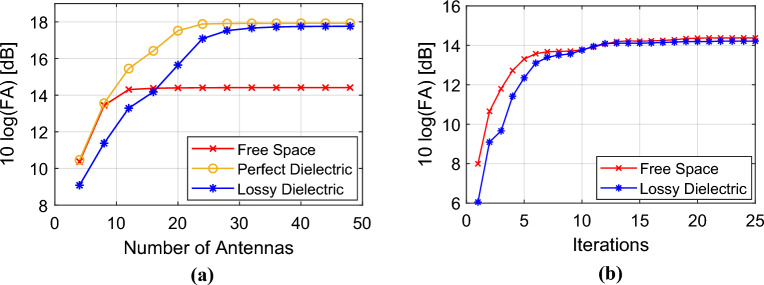
Variation of the FA parameter value for $f = 2.4\,{\rm GHz}$ and $|I_{{e_{i} }}| = 1$. (**a**) with respect to changing the number of antennas, in free space, perfect dielectric with $\varepsilon_{r} = 5.28$, and lossy dielectric with $\varepsilon_{r} = 5.28$, and $\tan \delta = 0.145$. (**b**) with respect to optimization iterations, for *N*=16 in free space and perfect dielectric with $\varepsilon_{r} = 5.28$.

Utilizing the full wave method for optimization demands a substantial amount of time. In contrast, leveraging the proposed theoretical analysis leads to a significant reduction in optimization time. According to the given numbers in Table 4, the proposed method is almost 80 times faster than the CST full-wave optimization method. The value of FA by increasing the number of iterations is shown in Fig. 7b.

**Table 4:** The phase optimization time and iterations.

Medium type		Lossy dielectric ($\varepsilon_{r} = 5.28, \, \tan \delta = 0.145$)
CST optimization	Time per iteration [min]	13.37
	Number of iterations	More than 400
MATLAB optimization (proposed method)	Time per iteration [min]	1.24
	Number of iterations	25

The simulations have been performed using a processing system with an Intel(R) Core (TM) i7-7500U CPU @ 2.70GHz and 12.0 GB RAM.

### Medium type and number of antennas

As depicted in Fig. 7a, the value of FA increases with the growing number of antennas; however, this value eventually saturates after reaching a certain number of antennas. Consequently, the optimal number of antennas can be determined based on the FA value for the desired optimal focusing. Moreover, Fig. 7 illustrates the impact of the medium type on the variations in the FA value.

## Conclusion

In this paper, a theoretical analysis and complete investigation of near-field electromagnetic power for a circumferential array of linear electrical sources are presented. The FA parameter is introduced to evaluate a method’s capacity for power focusing, facilitating the realization of two-dimensional power focusing. To achieve power focusing at the desired location, the excitation phases of the antennas are initially optimized to maximize the FA parameter. In the second stage of optimization, the excitation amplitudes of the antennas are determined to maximize the power at the focusing point while minimizing the level of side lobes. The results indicate that the behavior of the circumferential array using electric linear source elements closely aligns with the behavior of this array using half-wave dipole antenna elements when utilizing the same excitations for both arrays. Indeed, as observed in the CST full-wave results, employing the excitations derived from the proposed method for the half-wave dipole antenna array enables the achievement of power focusing using this array. Furthermore, the utilization of the proposed method enables faster acquisition of excitations compared to the full wave method, while the accuracy is maintained. As observed, increasing the number of antennas within a fixed study area enables more precise focusing and reduction of side lobe levels relative to the power value at the focusing point. The FA parameter can increase in dielectric and lossy mediums. Additionally, the FA parameter increases with the increasing number of antennas, allowing for the selection of an appropriate number of antennas based on the FA value.

### Supplementary Information

Supplementary Information.

## Data Availability

The dataset generated and/or the analysis performed in this study are available from the corresponding author upon a reasonable request.
