# Supplementary material for: Homogeneous microwave near-field power focusing using a cylindrical antenna array
Source: Sci Rep. 2023 Sep 7;13:14698. doi: 10.1038/s41598-023-41866-z (PMC10484950; doi:10.1038/s41598-023-41866-z)
Supplement: Supplementary file 1 — Supplementary Information. [file 41598_2023_41866_MOESM1_ESM.docx]

**Appendix (1).** Obtaining $\hat{a}_{\psi_{i}}$:

, $\hat{a}_{\rho'_{i}}=\frac{r'}{\left|r'\right|}=\frac{r-r_{s_{i}}}{\left|r-r_{s_{i}}\right|}$

$$\begin{matrix} \left(\alpha_{i}\hat{a}_{x}+\beta_{i}\hat{a}_{y}\right).\left(\frac{\left(x-x_{s_{i}}\right)\hat{a}_{x}+\left(y-y_{s_{i}}\right)\hat{a}_{y}}{\left|\left(x-x_{s_{i}}\right)\hat{a}_{x}+\left(y-y_{s_{i}}\right)\hat{a}_{y}\right|}\right)=0\Rightarrow\left(\alpha_{i}\hat{a}_{x}+\beta_{i}\hat{a}_{y}\right).\left(\frac{\left(x-x_{s_{i}}\right)\hat{a}_{x}+\left(y-y_{s_{i}}\right)\hat{a}_{y}}{\sqrt{\left(x-x_{s_{i}}\right)^{2}+\left(y-y_{s_{i}}\right)^{2}}}\right)=0 \\ \Rightarrow\frac{\left(x-x_{s_{i}}\right)\alpha_{i}+\left(y-y_{s_{i}}\right)\beta_{i}}{\sqrt{\left(x-x_{s_{i}}\right)^{2}+\left(y-y_{s_{i}}\right)^{2}}}=0\Rightarrow\alpha_{i}=-\frac{\left(y-y_{s_{i}}\right)}{\left(x-x_{s_{i}}\right)}\beta_{i} \end{matrix}$$

$$\left|\alpha_{i}\hat{a}_{x}+\beta_{i}\hat{a}_{y}\right|=1\text{ }\Rightarrow\text{ }\alpha_{i}^{2}+\beta_{i}^{2}=1\Rightarrow\left(-\frac{\left(y-y_{s_{i}}\right)}{\left(x-x_{s_{i}}\right)}\beta_{i}\right)^{2}+\beta_{i}^{2}=1$$

$$\begin{matrix} \beta_{i}=\frac{\left(x-x_{s_{i}}\right)}{\sqrt{\left(x-x_{s_{i}}\right)^{2}+\left(y-y_{s_{i}}\right)^{2}}},\text{ }\Rightarrow\text{ }\beta_{i}=\frac{\left(x-x_{s_{i}}\right)}{R_{i}} \\ \alpha_{i}=-\frac{\left(y-y_{s_{i}}\right)}{\sqrt{\left(x-x_{s_{i}}\right)^{2}+\left(y-y_{s_{i}}\right)^{2}}},\text{ }\Rightarrow\text{ }\alpha_{i}=-\frac{\left(y-y_{s_{i}}\right)}{R_{i}} \end{matrix}$$

**Appendix (2).** Obtaining the Poynting vector for the given problem structure.

$$S=\frac{1}{2}\left(\sum_{j=1}^{N} E_{z_{j}}\hat{a}_{z}\right)\times\left(\sum_{i=1}^{N} H_{\psi_{i}}\hat{a}_{\psi}\right)^{*}$$

$$\Rightarrow S=\frac{1}{2}\left(-\frac{k^{2}}{4\omega\varepsilon}\left(\sum_{j=1}^{N} I_{e_{j}}H_{0}^{(2)}(kR_{j})\right)\hat{a}_{z}\right)\times\left(\sum_{i=1}^{N} H_{\psi_{i}}\left(\alpha_{i}\hat{a}_{x}+\beta_{i}\hat{a}_{y}\right)\right)^{*}$$

$$S=-\frac{k^{2}}{8\omega\varepsilon}\left(\left(\sum_{j=1}^{N} \sum_{i=1}^{N} I_{e_{j}}H_{0}^{(2)}(kR_{j})\left(H_{\psi_{i}}\alpha_{i}\right)^{*}\right)\hat{a}_{y}-\left(\sum_{j=1}^{N} \sum_{i=1}^{N} I_{e_{j}}H_{0}^{(2)}(kR_{j})\left(H_{\psi_{i}}\beta_{i}\right)^{*}\right)\hat{a}_{x}\right)$$

$$S=-\frac{k^{2}}{8\omega\varepsilon}\left(\left(\sum_{j=1}^{N} \sum_{i=1}^{N} I_{e_{j}}H_{0}^{(2)}(kR_{j})\left(-j\frac{k}{4}I_{e_{i}}H_{1}^{(2)}(kR_{i})\alpha_{i}\right)^{*}\right)\hat{a}_{y}-\left(\sum_{j=1}^{N} \sum_{i=1}^{N} I_{e_{j}}H_{0}^{(2)}(kR_{j})\left(-j\frac{k}{4}I_{e_{i}}H_{1}^{(2)}(kR_{i})\beta_{i}\right)^{*}\right)\hat{a}_{x}\right)$$

$$S=-j\frac{k^{2}}{32\omega\varepsilon}\left(\left(\sum_{j=1}^{N} \sum_{i=1}^{N} I_{e_{j}}I_{e_{i}}^{*}k^{*}H_{0}^{(2)}(kR_{j})\left(H_{1}^{(2)}(kR_{i})\right)^{*}\alpha_{i}\right)\hat{a}_{y}-\left(\sum_{j=1}^{N} \sum_{i=1}^{N} I_{e_{j}}I_{e_{i}}^{*}k^{*}H_{0}^{(2)}(kR_{j})\left(H_{1}^{(2)}(kR_{i})\right)^{*}\beta_{i}\right)\hat{a}_{x}\right)$$

$$S=j\frac{k^{2}}{32\omega\varepsilon}k^{*}\left(\left(\sum_{j=1}^{N} \sum_{i=1}^{N} I_{e_{j}}I_{e_{i}}^{*}H_{0}^{(2)}(kR_{j})\left(H_{1}^{(2)}(kR_{i})\right)^{*}\beta_{i}\right)\hat{a}_{x}-\left(\sum_{j=1}^{N} \sum_{i=1}^{N} I_{e_{j}}I_{e_{i}}^{*}H_{0}^{(2)}(kR_{j})\left(H_{1}^{(2)}(kR_{i})\right)^{*}\alpha_{i}\right)\hat{a}_{y}\right)$$

$$\begin{matrix} S_{x}=j\frac{k^{2}}{32\omega\varepsilon}k^{*}\sum_{j=1}^{N} \sum_{i=1}^{N} I_{e_{j}}I_{e_{i}}^{*}H_{0}^{(2)}(kR_{j})\left(H_{1}^{(2)}(kR_{i})\right)^{*}\beta_{i} \\ S_{y}=-j\frac{k^{2}}{32\omega\varepsilon}k^{*}\sum_{j=1}^{N} \sum_{i=1}^{N} I_{e_{j}}I_{e_{i}}^{*}H_{0}^{(2)}(kR_{j})\left(H_{1}^{(2)}(kR_{i})\right)^{*}\alpha_{i} \end{matrix}$$

For free space:

$$S=j\frac{k^{3}}{32\omega\varepsilon}\left(\left(\sum_{j=1}^{N} \sum_{i=1}^{N} I_{e_{j}}I_{e_{i}}^{*}H_{0}^{(2)}(kR_{j})H_{1}^{(1)}(kR_{i})\beta_{i}\right)\hat{a}_{x}-\left(\sum_{j=1}^{N} \sum_{i=1}^{N} I_{e_{j}}I_{e_{i}}^{*}H_{0}^{(2)}(\beta R_{j})H_{1}^{(1)}(\beta R_{i})\alpha_{i}\right)\hat{a}_{y}\right)$$

$$\begin{matrix} S_{x}=j\frac{k^{3}}{32\omega\varepsilon}\sum_{j=1}^{N} \sum_{i=1}^{N} I_{e_{j}}I_{e_{i}}^{*}H_{0}^{(2)}(kR_{j})H_{1}^{(1)}(kR_{i})\beta_{i} \\ S_{y}=-j\frac{k^{3}}{32\omega\varepsilon}\sum_{j=1}^{N} \sum_{i=1}^{N} I_{e_{j}}I_{e_{i}}^{*}H_{0}^{(2)}(kR_{j})H_{1}^{(1)}(kR_{i})\alpha_{i} \end{matrix}$$

**Appendix (3).** Obtaining the $p_{f}$for the given problem structure.

For free space:

$$p_{f}=j\frac{k^{3}}{32\omega\varepsilon}\sum_{j=1}^{N} \sum_{i=1}^{N} \left(I_{e_{j}}I_{e_{i}}^{*}\left(kH_{0}^{(1)}(kR_{i})H_{0}^{(2)}(kR_{j})-kH_{1}^{(2)}(kR_{j})H_{1}^{(1)}(kR_{i})\left(\beta_{j}\beta_{i}+\alpha_{j}\alpha_{i}\right)\right)\right)$$

**Appendix (4).** Details of the genetic algorithm used in the article.

Genetic algorithm optimization for continuous-variable

Number of variables=*N*

Population Size=20;

Crossover rate=0.9;

Mutation rate=0.3;
